# Tumor-expressed adrenomedullin accelerates breast cancer bone metastasis

**DOI:** 10.1186/s13058-014-0458-y

**Published:** 2014-12-02

**Authors:** Valerie A Siclari, Khalid S Mohammad, Douglas R Tompkins, Holly Davis, C Ryan McKenna, Xianghong Peng, Lisa L Wessner, Maria Niewolna, Theresa A Guise, Attaya Suvannasankha, John M Chirgwin

**Affiliations:** 1 Department of Biochemistry and Molecular Genetics, University of Virginia, Charlottesville, 22908 VA USA; 2 Division of Endocrinology and Metabolism, Department of Medicine, University of Virginia, 450 Ray C Hunt Dr, Charlottesville, 22908 VA USA; 3 Division of Endocrinology, Department of Medicine, Indiana University School of Medicine, 980 Walnut, St, C321-C, Indianapolis, 46202 IN USA; 4 Richard L. Roudebush VA Medical Center, 1481 W 10th St, Indianapolis, 46202 IN USA; 5 Division of Hematology/Oncology, Department of Medicine, Indiana University School of Medicine, 980 Walnut St, C321-H, Indianapolis, 46202 IN USA

## Abstract

**Introduction:**

Adrenomedullin (AM) is secreted by breast cancer cells and increased by hypoxia. It is a multifunctional peptide that stimulates angiogenesis and proliferation. The peptide is also a potent paracrine stimulator of osteoblasts and bone formation, suggesting a role in skeletal metastases—a major site of treatment-refractory tumor growth in patients with advanced disease.

**Methods:**

The role of adrenomedullin in bone metastases was tested by stable overexpression in MDA-MB-231 breast cancer cells, which cause osteolytic bone metastases in a standard animal model. Cells with fivefold increased expression of AM were characterized *in vitro*, inoculated into immunodeficient mice and compared for their ability to form bone metastases versus control subclones. Bone destruction was monitored by X-ray, and tumor burden and osteoclast numbers were determined by quantitative histomorphometry. The effects of AM overexpression on tumor growth and angiogenesis in the mammary fat pad were determined. The effects of AM peptide on osteoclast-like multinucleated cell formation were tested *in vitro*. A small-molecule AM antagonist was tested for its effects on AM-stimulated *ex vivo* bone cell cultures and co-cultures with tumor cells, where responses of tumor and bone were distinguished by species-specific real-time PCR.

**Results:**

Overexpression of AM mRNA did not alter cell proliferation *in vitro*, expression of tumor-secreted factors or cell cycle progression. AM-overexpressing cells caused osteolytic bone metastases to develop more rapidly, which was accompanied by decreased survival. In the mammary fat pad, tumors grew more rapidly with unchanged blood vessel formation. Tumor growth in the bone was also more rapid, and osteoclasts were increased. AM peptide potently stimulated bone cultures *ex vivo*; responses that were blocked by small-molecule adrenomedullin antagonists in the absence of cellular toxicity. Antagonist treatment dramatically suppressed tumor growth in bone and decreased markers of osteoclast activity.

**Conclusions:**

The results identify AM as a target for therapeutic intervention against bone metastases. Adrenomedullin potentiates osteolytic responses in bone to metastatic breast cancer cells. Small-molecule antagonists can effectively block bone-mediated responses to tumor-secreted adrenomedullin, and such agents warrant development for testing *in vivo*.

**Electronic supplementary material:**

The online version of this article (doi:10.1186/s13058-014-0458-y) contains supplementary material, which is available to authorized users.

## Introduction

Adrenomedullin (AM) is a member of the calcitonin/amylin/calcitonin gene-related peptide (CGRP) family discovered in 1993. The 52–amino acid peptide is released from a larger precursor by posttranslational processing, along with a separate 20–amino acid proadrenomedullin peptide, PAMP [1]. Transcription of the gene is increased by the hypoxia-stabilized hypoxia-inducible factor 1α pathway [2]. AM is secreted into the circulation, where it binds to complement factor H [3]. The peptide signals via the calcitonin receptor-like receptor plus receptor activity–modifying protein (RAMP) 2 or 3, activating adenylate cyclase [4]. Responses to AM include vasodilation, bronchodilation, angiogenesis, lymphangiogenesis, inhibition of microbial growth, and regulation of renal function, hormone secretion, cell proliferation and apoptosis [5].

Many cancers, including those of the lung, prostate, colon, ovary and breast, express AM [6],[7] and its receptor [8]. Increased AM production by tumors and high concentrations of the peptide in plasma correlate with lymph node metastases in breast cancer patients, suggesting that AM contributes to an aggressive metastatic phenotype [9]. AM regulates cell proliferation, inhibits apoptosis and stimulates angiogenesis in cancer cells [6],[7],[10]. AM inhibition decreases subcutaneous tumor growth of glioblastoma, lung, colon, breast and pancreatic cancers in mice [11]-[14].

AM is also a potent stimulator of bone cells [15], which express both ligand and receptor [16]. Exogenous AM peptide potently stimulates new bone formation and osteoblast proliferation through a cAMP-dependent pathway [17],[18]. No effect of AM on bone-resorbing osteoclasts has been found [19], and a role for the peptide in bone metastases has not previously been tested.

More than 80% of breast cancers are positive for AM expression [9], whereas other tumor types that cause bone metastases [20] also express the peptide, which can act on both tumor and bone cells [21]. At least 80% of patients with advanced breast cancer have bone metastases, and survival from the time of first diagnosis of skeletal involvement is approximately 24 months [22],[23]. Bone metastases are incurable, and more effective treatments are needed [20],[23]. Because of its specific effects on bone and expression by the majority of tumors, we hypothesized that AM would be an enhancing rather than primary causal factor in metastases to bone, suggesting that AM could be a therapeutic target for treatment of bone metastases. Stable AM expression by MDA-MB-231 human breast cancer cells was experimentally increased fivefold to mimic the effect of hypoxia to induce AM [2]. The cells showed unchanged molecular parameters *in vitro* but increased bone metastases and mammary fat pad (MFP) growth *in vivo*. Small-molecule receptor antagonists effectively blocked the paracrine actions of AM on bone. In a novel *ex vivo* model of tumor growth in bone metastases, adding AM increased the growth of tumor in bone and stimulated expression of the osteoclast marker tartrate-resistant acid phosphatase (TRAP) only in the presence of tumor while changing the cell source of the osteoclast regulator, receptor activator of nuclear factor κB ligand (RANKL). The AM antagonist 16311 blocked the increases in RANKL and TRAP and decreased tumor growth in bone. The results suggest that small-molecule antagonists may be effective against breast cancer skeletal metastases by blocking the actions of AM to potentiate osteolytic responses of bone to tumor.

## Methods

### Plasmids

The complete 1,494-nucleotide human preproAM mRNA sequence [GenBank:BC015961] was released from the pOTB7 vector by EcoRI-PspOMI restriction enzyme digestion and ligated between the EcoRI and NotI sites of pIRESneo3 (Clontech Laboratories, Mountain View, CA, USA) to create pIRESneo3-hAM. In the vector, the cytomegalovirus (CMV) promoter drives transcription of a bicistronic mRNA encoding both preproAM and the neomycin resistance cassette, separated by an internal ribosome entry site (IRES), to facilitate antibiotic selection of AM-expressing clones. Restriction mapping with EcoRI and AciI confirmed the correct orientation of the AM insert relative to the CMV promoter. An emerald green fluorescent protein (emGFP) cassette from pLenti6.2 (Invitrogen, Carlsbad, CA, USA) was cloned into the EcoRV site of pIRESneo3 to create pIRESneo3-emGFP for use as the vector control.

### Adrenomedullin antagonists

Small-molecule antagonists of AM [24] were provided by Dr. Frank Cuttitta of the National Cancer Institute (NCI), National Institutes of Health (Bethesda, MD, USA). They were dissolved in dimethyl sulfoxide, diluted in phosphate-buffered saline (PBS), sterile-filtered and added to bone organ cultures at the indicated final concentrations. NSC 16311 is 2-(1-ethyl-4-hydroxy-4-piperidyl)-2-phenyl acetic acid (CAS 5449-34-3); NSC 37133 is 2-[(4-carboxyphenyl)methyl]benzoic acid (CAS 6268-08-2); and NSC 28086 is 2-hydroxy-2,2-bis(4-phenylphenyl)-acetic acid (CAS 6334-91-4).

### Cell culture

MDA-MB-231 cells were purchased from the American Type Culture Collection (Manassas, VA, USA) and have been previously characterized for their behavior in a model of bone metastasis [25]. MDA-MB-231 cells were cultured in Dulbecco’s modified Eagle’s medium (DMEM; Mediatech, Manassas, VA, USA) supplemented with 10% fetal bovine serum (FBS; Atlanta Biologicals, Norcross, GA, USA) and 1% penicillin/streptomycin. The stable pools and single-cell clones were grown in DMEM and 10% FBS plus G418 (1,100 μg/ml and 150 μg/ml, respectively). Cells were incubated at 37°C and 5% CO_2_ in a humidified incubator.

### Isolation of stable clones

An aggressive bone metastatic variant of the human breast cancer line MDA-MB-231 [25] was transfected with either pIRESneo3-hAM or pIRESneo3-emGFP control using FuGENE HD transfection reagent (Promega, Madison, WI, USA). Cells were selected with G418 to create a stable pool. Clones were isolated by limiting dilution in the presence of antibiotics. Increased AM mRNA was assayed by real-time PCR. Green fluorescent protein (GFP) expression in control transfectants was confirmed by fluorescence microscopy. Clones were cultured for 60 days in the absence of G418 selection and retested for AM and GFP expression to assure phenotypic stability. Two stable GFP and two AM-overexpressing clones with similar characteristics *in vitro* were chosen for use *in vivo* to exclude response due to clonal variability.

### Detection of secreted human peptides

MDA-MB-231 parental cells—two GFP- and two AM-overexpressing clones—were plated at 10^6^ cells per 145-mm dish and grown to >90% confluence. Cells were rinsed with 1× PBS and then grown in 10 ml of 0.1% bovine serum albumin and 1% penicillin/streptomycin in DMEM for 24 hours. Media were collected, mixed with protease inhibitors (aprotinin, phenylmethylsulfonyl fluoride and leupeptin), centrifuged to remove debris and assayed for human AM and PAMP peptides with enzyme immunoassay kits (Phoenix Pharmaceuticals, Burlingame, CA, USA).

### Determination of growth *in vitro*

Cells were plated at 2,500 cells per well in a 96-well plate on day 0. 3-(4,5-dimethylthiazol-2-yl)-2,5-diphenyltetrazolium bromide (MTT) assays were performed on days 1, 3 and 5 per the manufacturer’s instructions (Promega). Optical density was read at 570 nm (Synergy HT plate reader with KC4 v3.1 software; BioTek, Winooski, VT, USA). All values were normalized to the value at day 1 to control for initial plating differences.

### Animal experiments

The animal protocols were approved by the Institutional Animal Care and Use Committee at the University of Virginia and were in accordance with the US Public Health Service Policy on Humane Care and Use of Laboratory Animals and the US Animal Welfare Act. Four-week-old athymic nude mice were purchased from Harlan Laboratories (Indianapolis, IN, USA) and housed under barrier conditions. The mice were killed whenever established criteria for pain and discomfort—in particular, cachexia or spinal paralysis, or tumor burden—were reached.

### Bone metastasis model

Tumor cells were trypsinized, washed twice and resuspended at 10^6^ cells/ml in PBS. The mice were anesthetized using ketamine and xylazine. Intracardiac inoculations of 10^5^ cells were performed by percutaneous injection into the left cardiac ventricle of 5-week-old female nude mice (*n* = 12 per cell line) using a 26-gauge needle [25]. The MDA-MB-231 breast cancer cells used *in vivo* cause reproducible metastasis to the appendicular skeleton in 100% of inoculated mice, visible on X-rays by 3 weeks. This is the most commonly used model of skeletal metastasis, and it mimics the final stages of colonization of bone and growth of lesions to detectable size. As such, it has been used extensively for identification of preclinical targets and drug candidates. The breast cancer cells do not metastasize from the primary site and do not cause lung metastases, although other sublines of MDA-MB-231 do so [26]. All mice were inspected *post mortem* for metastases outside of bone. None were found.

### Radiography and analysis of osteolytic lesions

The mice were X-rayed weekly starting at 2 weeks after tumor cell inoculation with an MX-20 digital radiography system and digital camera (Faxitron Bioptics, Tucson, AZ, USA). Each mouse was X-rayed at 1× magnification in prone and lateral positions. Magnified images (4× magnification) of suspected lesions were obtained. Osteolytic lesion areas were analyzed on digital images using MetaMorph software (Molecular Devices, Sunnyvale, CA, USA).

### Bone histology and computerized quantitative histomorphometry

Hind limbs were fixed for 48 hours in 10% neutral buffered formalin, decalcified in 10% ethylenediaminetetraacetic acid for 2 weeks, processed and embedded in paraffin wax. An automated microtome (HM 355S; MICROM International, Walldorf, Germany) was used to cut 3.5-μm-thick, longitudinal, midsagittal sections of the embedded tissue. Sections were stained with hematoxylin and eosin (H&E) and TRAP (for the identification of osteoclasts) for histomorphometric analysis. Images were captured with a Leica DM LB compound microscope (Leica Microsystems, Buffalo Grove, IL, USA) and a QImaging MicroPublisher cooled charge-coupled device color digital camera (QImaging, Surrey, BC, Canada). MetaMorph software was used to analyze total tumor burden and osteoclast number in multiple sections from the femora and tibiae of the hind limbs. Tumor burden was defined as the area of bone occupied by the tumor at 50× magnification of H&E-stained sections [27]. Osteoclast number per millimeter of bone surface at the interface with tumor was measured from TRAP-stained tibiae at 200× magnification.

### Mammary fat pad model

Tumor cells were inoculated into the pectoral MFPs of 5-week-old female nude mice using a 27-gauge needle (two MFPs/mouse; ten mice/cell line). A total of 10^6^ cells in 100 μl of PBS were injected per MFP. Tumors were measured by caliper three times per week. Tumor burden was calculated by using the following formula: tumor volume = 4/3π × *L*/2 × (*w*/2)^2^, where L = midaxis length and *w* = midaxis width.

### CD31 immunohistochemistry

Immunohistochemical analyses were performed on formalin-fixed, paraffin-embedded MFP sections. Rat anti-mouse CD31 antibody (catalog no. 550274) was purchased from BD Pharmingen (San Jose, CA, USA). Proteinase K was used for antigen retrieval. Ten percent normal rabbit serum in PBS was used as the blocking agent. The secondary antibody, biotinylated rabbit anti-rat immunoglobulin G, was obtained from Chemicon International (Temecula, CA, USA). Antibody binding was visualized using VECTASTAIN Elite ABC kits (Vector Laboratories, Burlingame, CA, USA) and diaminobenzidine kits (DakoCytomation, Glostrup, Denmark). Samples were counterstained with hematoxylin. CD31-stained vessels were counted as described elsewhere [28] in two 200× magnified images for four tumors for each cell line.

### Osteoclast formation *in vitro*

Bone marrow was flushed from 5-week-old C57BL/6 mice and plated at 1.9 × 10^6^ cells/well in a 48-well plate, along with 5 × 10^4^ primary osteoblasts derived from neonatal mouse calvariae by multiple digestions with dispase and collagenase. Bone marrow and/or osteoblast co-cultures were grown in α-minimal essential medium supplemented with 10% FBS and 1% penicillin/streptomycin. Cells were treated for 6 days with medium alone, 10^−7^ M AM (Bachem, Bubendorf, Switzerland), 10^−7^ M PAMP (Bachem) or 10^−8^ M 1,25-dihydroxyvitamin D_3_ (Enzo Life Sciences, Farmingdale, NY, USA). Cells were fixed with citrate/acetone/formaldehyde solution and stained for TRAP using a leukocyte acid phosphatase kit (Sigma Diagnostics, St Louis, MO, USA). Cells that stained positive for TRAP with three or more nuclei were counted and compared [29].

### *Ex vivo*bone organ cultures

Neonatal mouse calvarial bone explants were trimmed and cultured for 7 days in serum-free BGJb medium (Life Technologies, Carlsbad, CA, USA), which was changed every 48 hours, in the presence or absence of 100 nM AM. New bone formation was determined by quantitative histomorphometry of decalcified sections [30]. Primary osteoblasts were isolated from the same neonatal calvarial source [31].

### *Ex vivo*growth of tumor with bone

Segments of neonatal mouse bone were prepared by using a modification of the method described by Mohammad *et al.* [30]. Calvariae from killed 11-day-old C57BL/6 mouse pups were cut into 3-mm disks with a biopsy punch and placed into wells of uncoated 24-well plates. Breast cancer cells (5 × 10^3^) were added to 0.5 ml of BGJb medium supplemented with 20% fetal calf serum, 500 nM human insulin and 100 μM ascorbic acid. Plates were incubated at 37°C in 5% CO_2_. The medium was changed every 2 to 3 days. At day 6, bones were washed in PBS, placed in 1.0 ml of QIAzol lysis reagent (QIAGEN, Valencia, CA, USA) and homogenized for 1 minute with zirconium beads in a BeadBug microtube homogenizer (Benchmark Scientific, South Plainfield, NJ, USA) at 280 strokes/min according to the manufacturer’s instructions. Aqueous phase RNA was isolated using an RNeasy Mini Kit (QIAGEN). First-strand cDNA for PCR was made with an iScript cDNA synthesis kit (Bio-Rad Laboratories, Hercules, CA, USA). Breast cancer cells were transduced with lentiviral particles from GenTarget (San Diego, CA, USA), according to the manufacturer’s instructions, with a vector encoding secreted *Cypridina* luciferase driven by a CMV promoter, plus a red fluorescent protein (RFP)-blasticidin fusion protein cassette for selection. RFP-positive MDA-MB-231 cells were isolated by fluorescence-activated cell sorting. Secretion of luciferase was used as an indicator of tumor burden [32] by assay of conditioned media with a BioLux *Cypridina* luciferase assay kit obtained from New England BioLabs (Ipswich, MA, USA) and a Turner BioSystems TD 20/20 luminometer (Promega). The results were expressed as relative luminescence units.

### PCR experiments

Total RNA was isolated using QIAGEN RNeasy Mini Kits including treatment with DNase. RNA was converted to cDNA using Omniscript RT Kits from QIAGEN with 16-mer oligo(dT) primers and then analyzed by PCR with an Eppendorf Mastercycler (Eppendorf, Hamburg, Germany) or by quantitative PCR using the QuantiTect SYBR Green PCR Kit (QIAGEN) and an iCycler Single-Color Real-Time Detection System (Bio-Rad Laboratories). Human AM mRNA was detected using these primers: forward, 5′-GGA-AGA-GGG-AAC-TGC-GGA-TGT-3′; reverse, 5′-GGC-ATC-CGG-ACT-GCT-GTC-T-3′. To normalize the data, human ribosomal protein L32 (RPL32) mRNA was amplified with the following primers: forward, 5′-CAG-GGT-TCG-TAG-AAG-ATT-CAA-GGG-3′; reverse, 5′-CTT-GGA-GGA-AAC-ATT-GTG-AGC-GAT-C-3′. Relative mRNA expression was calculated using the 2^−∆∆CT^ method [33]. Species-specific PCR primers were designed with the online Primer3 tool [34] and tested for species specificity with the National Center for Biotechnology Information (NCBI) primer design tool [35] by Blast searching against the targeted sequence and versus both *Mus musculus* and *Homo sapiens* sequence databases to eliminate cross-species and erroneous amplifications and allowing amplification of transcript variants. The templates used for primer design were GenBank RefSeq files for the designated genes. The NCBI primer design tools proved unsatisfactory for the initial design of primers suitable for species-specific amplification. Primers were then separately tested using real-time PCR with cDNAs from mouse calvariae and human tumor cells. Primer sets were accepted only when they yielded results on the proper species and gave single melt curve peaks, a cycle efficiency >95%, threshold cycle values <33 and only one band of DNA of the appropriate size on gel electrophoresis. All PCR data were normalized to the ribosomal large subunit protein 32 housekeeping control, *RPL32*, specific to mice or humans [36]. A lower case *m* or *h* in front of gene symbols indicates mouse or human specificity. Additional species-specific primer sequences can be found in the Additional file 1.

### Statistical analyses

Data were compiled in Microsoft Excel files (Microsoft, Redmond, WA, USA) and analyzed with GraphPad Prism software (GraphPad Software, La Jolla, CA, USA). Two-way analysis of variance (ANOVA) with the Bonferroni posttest was used to determine differences between osteolytic lesion area, MFP tumor volume and proliferation *in vitro*. One-way ANOVA with Tukey’s multiple-comparisons posttest was used for histomorphometric analysis to determine differences in tumor area and osteoclast number per millimeter of tumor–bone interface, osteoclast number in the bone marrow/osteoblast co-cultures, number of CD31+ vessels, bone formation in calvarial cultures and changes in gene expression in tumor/bone co-cultures. Logrank analysis was used to assess differences between survival curves. The results are shown with mean ± standard deviation and statistical significance levels of *P < 0.05, **P < 0.01, ***P < 0.001. Significance is in comparison to controls in all figures, except where stated otherwise.

## Results and discussion

### Breast cancer cell lines that metastasize to bone express adrenomedullin

**Figure 1:**
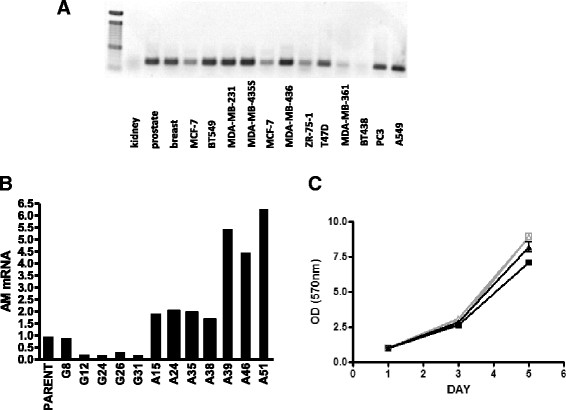
**Adrenomedullin expression by breast cancer cell lines and effects of adrenomedullin on tumor and bone cells. (A)** Adrenomedullin (*AM) RNA in human breast cancer cell lines* that metastasize to bone. PCR detection of human AM mRNA in from left to right: normal tissues (kidney, prostate and breast), breast cancer cell lines (MCF-7, BT549, MDA-231, MCF-7, MDA-MB-436, ZR-75-1, T47D, MD-MB-361 and BT438) and three additional cancer cell lines that cause osteolytic bone metastases: MDA-MB-435S melanoma cells, PC-3 prostate cancer cells and A549 lung adenocarcinoma cells. cDNA (200 ng) was amplified for 30 cycles. The PCR product is 600 bp. It is unclear if kidney expresses only low amounts of AM mRNA or if the RNA was degraded. **(B)** *Stable overexpression* of human AM mRNA in MDA-MB-231 cells. Clones were tested for stable human AM mRNA expression using real-time PCR after 60 days of culture in the absence of antibiotic selection. G8-G31 are MDA-MB-231 control clones, and A15-A51 are MDA-MB-231 clones overexpressing AM. **(C)** *Proliferation in vitro* of breast cancer cells. Proliferation of two AM-overexpressing clones (A39, gray triangle; A51, black triangle) and two control clones (G8, gray square; G26, black square) were compared using a 3-(4,5-dimethylthiazol-2-yl)-2,5-diphenyltetrazolium bromide (MTT; tetrazolium blue dye) assay. Proliferation was compared after 1, 3 and 5 days of growth. OD, Optical density.

Human breast cancer cell lines previously described to metastasize to bone in mice following intracardiac inoculation [27] were surveyed for AM mRNA. The majority of breast cancer lines were positive, including the osteolytic lines MDA-MB-231 and BT549; the osteoblastic lines ZR75, MCF-7 and T47D; and MDA-MB-436 and MDA-MB-361, which do not metastasize to bone. Only BT438 lacked detectable AM mRNA. Other human tumors metastatic to bone expressed AM, including PC-3 prostate cancer cells, A549 lung cancer cells and MDA-MB-435 melanoma cells. Normal tissues expressed AM, including breast, kidney and prostate (Figure 1A). The data do not suggest that AM expression contributes to specific tropism to bone characteristics of certain tumors.

Because of its potent stimulatory activity toward osteoblasts, AM could contribute to the growth of tumors that have already metastasized to skeletal sites. First, we tested the effects of increased AM expression on the progression of osteolytic bone metastases caused by MDA-MB-231 cells, which were chosen because low-expressing lines such as MCF7 and T47D show substantially altered phenotype and growth *in vitro* when transfected with AM [37] and cause more indolent bone metastases than MDA-MB-231 cells [25], which are the standard and most widely used model of skeletal metastases [38]. Tumor growth was followed in the MFP in a separate experiment to control for *in vivo* effects of AM not specific to bone.

### Generation of MDA-MB-231 clones with fivefold increased expression of adrenomedullin

Cells were transfected with either the pIRESneo3-hAM or pIRESneo3-emGFP plasmid. emGFP expression was used for the control to minimize differences in growth rate of transfectants often encountered after selection for antibiotic resistance. Single-cell clones were grown for 60 days without antibiotics to test for stable overexpression. We selected two clones with stable fivefold overexpression of human AM mRNA (Figure 1B) and five clones with stable GFP expression detected by fluorescence microscopy. The proadrenomedullin precursor encodes PAMP in addition to AM. The PAMP concentration in media conditioned by the tumor cells was less than the limit of detection of the assay, 0.08 ng/ml, perhaps due to proteolytic degradation of the peptide. Tumor-secreted AM peptide concentrations ranged from 0.9 to 2.5 ng per 10 million cells per 24 hours by enzyme immunoassay in media conditioned by the cell lines, but did not correlate with mRNA levels. MDA-MB-231 cells express complement factor H (data not shown), which binds AM with nanomolar affinity, interfering with detection of the peptide [3].

Two AM-overexpressing clones (A39 and A51) and two GFP control clones (G8 and G26) were used for further experimentation (Figure 1B). The members of each pair were compared to ensure that responses were not due to clonal variation. The four clones showed no significant growth differences *in vitro* as measured by MTT assay (Figure 1C). Overexpression of AM in T47D breast cancer cell cells did not alter proliferation *in vitro*, unless the cells were grown serum-free [37]. AM overexpression in T47D cells increased the percentage of cells in sub-G_0_/G_1_-phase and decreased the percentage in S-phase. AM overexpression in MDA-MB-231 cells did not alter progression through the cell cycle as determined by flow cytometric analysis of propidium iodide–stained cells (data not shown) or expression (analyzed by PCR) of a panel of tumor-secreted factors important in bone metastasis [20],[39]: interleukin 11 (IL-11), PTHrP, IL-8, cysteine-rich protein 61, connective tissue growth factor and vascular endothelial growth factor (data not shown).

### Adrenomedullin overexpression increased osteolytic bone metastases

**Figure 2:**
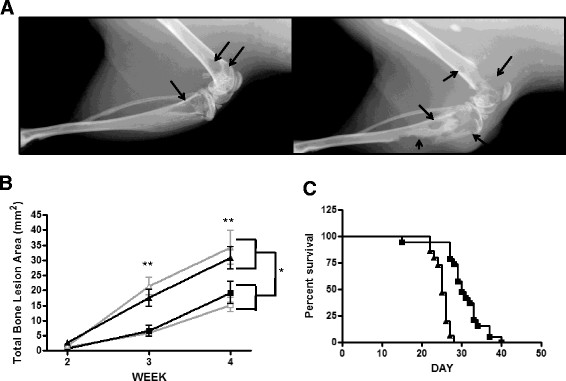
**Adrenomedullin overexpression increases osteolytic bone destruction and decreases survival in mice.** Two control (G8 and G26) and two adrenomedullin (AM)-overexpressing (A39 and A51) MDA-MB-231 clones were injected into the left cardiac ventricle of female athymic nude mice (*n* = 12/cell line). **(A)** *Representative radiographs* of the bone destruction due to control (*left panel*) and AM-overexpressing clones (*right panel*) at 4 weeks after tumor cell inoculation. Original magnification, ×4. Arrows point to osteolytic lesions. **(B)** *Quantitative radiographic lesion analysis* of MDA-MB-231 control and AM+ clones over time (G8, gray square; G26, black square; A39, gray triangle; A51, black triangle). *P < 0.05, **P < 0.01. **(C)** *Mouse survival.* The two control clones (G8 and G26, black squares) and the two AM+ clones (A39 and A51, black triangles) were grouped together in the analysis.

Nude mice inoculated with MDA-MB-231 cells by the intracardiac route reproducibly develop metastases to the appendicular skeleton, characterized by radiolucent regions visible on X-rays, indicating destruction of bone by tumor-associated osteoclasts. The cells rarely colonize to other sites, and the animals develop paraplegia and cachexia requiring that they be killed 4 weeks after inoculation [25],[38]. Two AM-overexpressing, two GFP control clones and parental cells were injected into the left cardiac ventricle of female nude mice. AM-overexpressing clones (Figure 2A, right panel) caused a significantly increased total osteolytic lesion area (indicated by arrows pointing to radiolucent regions) in bone compared to either GFP control cells (Figure 2A, left panel) or parental cells as measured by X-ray (19.3 mm^2^ vs. 6.1 mm^2^ at 3 weeks, 32.5 mm^2^ vs. 16.9 mm^2^ at 4 weeks; *P* < 0.001 for comparison of AM-overexpressing clones versus GFP control clones grouped together) (Figure 2B). The GFP control clones produced total osteolytic lesion areas similar to each other and the parental cell line (*P* = not significant). The AM-overexpressing clones produced total bone osteolytic lesion areas similar to each other (*P* = not significant). We inspected the histological sections for evidence of osteoblastic lesions because AM stimulates new bone formation, but we found no regions of clear osteoblastic reaction to tumor. Changes in osteoblasts are seldom seen in models of skeletal metastases, and methods for their quantification are lacking. We found no gross evidence of metastases to soft tissue sites in any of the mice. AM overexpression significantly decreased the overall survival of the mice from 30 to 25 days (Figure 2C).

**Figure 3:**
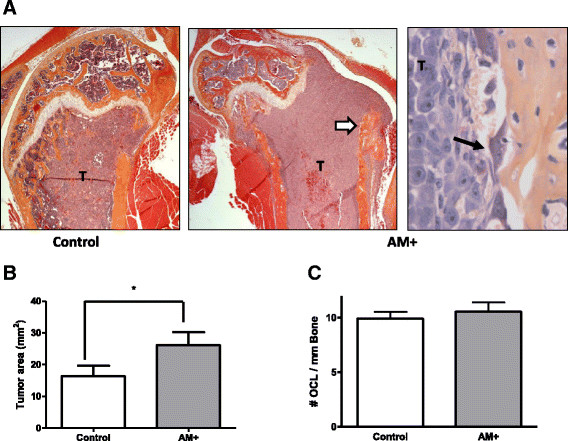
**Histomorphometric analysis of bone metastases. (A)** *Representative histological images* of mouse femoral sections. *Left* image represents the control group, and *middle* image represents the adrenomedullin (AM)-overexpressing group (hematoxylin and eosin stain; original magnification, ×31.25). *Right image* is a magnified region of an area in the AM-overexpressing bone metastasis that has osteolytic bone destruction tartrate-resistant acid phosphatase (TRAP) stain; original magnification, ×400). White arrow in middle image points to the magnified region shown in the right image. Tumor is labeled “T.” Black arrow points to a TRAP-positive (TRAP+; red) osteoclast along the tumor–bone interface. **(B)** *Tumor burden in bone.* Quantitative histomorphometric analysis of tumor area in the legs (tibia and femur). The two control (G8 and G26) and the two AM-overexpressing clones (A39 and A51) are grouped together in the graph. *P < 0.05. **(C)** *Osteoclast numbers.* TRAP+ osteoclasts along the bone–tumor interface were counted. There was no significant difference between the groups.

Tumor-bearing limbs were examined histologically. Figure 3A shows representative lesions, with a control tumor (T) shown in the left panel, a large AM-overexpressing tumor depicted in the center panel adjacent to bone (white arrow) and a region magnified in the right panel, showing a multinucleated osteoclast (dark arrow) at the tumor–bone interface. In the left and center panels, tumor has filled the marrow cavity below the growth plate, destroying trabecular bone and eroding endocortical bone. These features are typically seen in this model [25],[38]. Total tumor area in the legs, determined by quantitative histomorphometry, was almost doubled (*P* < 0.05) when the tumor overexpressed AM (Figure 3B). This determination was necessary because the osteolytic lesion area shown on the X-ray (Figure 2B) is not a direct measure of tumor burden. The number of bone-resorbing cells along the tumor–bone interface was also determined histomorphometrically. Multinucleated osteoclasts were identified by staining for the marker TRAP. The AM-overexpressing group had a number of osteoclasts along the tumor–bone interface similar to those of the GFP control groups (Figure 3C). The AM-overexpressing group was analyzed sooner after tumor inoculation than the GFP control group because of differences in survival. Thus, AM overexpression reduced the time by 5 days for tumors to stimulate formation of equivalent numbers of osteoclasts compared to GFP control tumors. Because this was a survival experiment, it was not possible to analyze osteoclast numbers earlier during the process of bone destruction, when they could be increased by AM versus controls.

**Figure 4:**
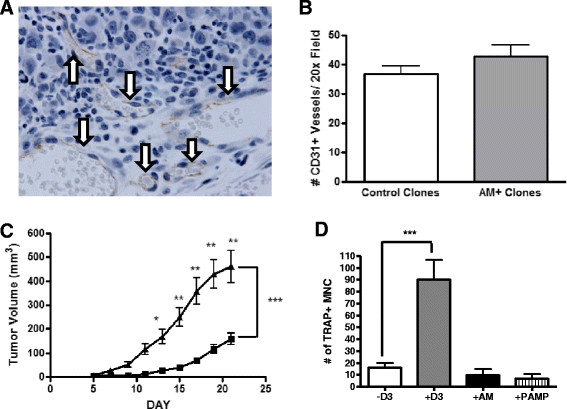
**Adrenomedullin did not affect tumor vascularization or osteoclastogenesis but increased tumor growth in the mammary fat pad. (A)** and **(B)** show tumor vascularization. **(A)** Representative mammary fat pad tumor section stained for CD31. Arrows point to vessels with brown immunostaining (original magnification, ×640). **(B)** Quantification of CD31+ vessels in tumor. Fields containing stained vessels were counted per ×200 magnification field (two fields per tumor, four tumors per cell line). There was no significant difference between the groups. **(C)** *Mammary fat pad growth.* Two control (G8 and G26) and two adrenomedullin (AM)-overexpressing (A39 and A51) MDA-MB-231 clones were injected into the mammary fat pad of female athymic nude mice (*n* = 10 mice/cell line; *n* = 2 mammary fat pads/mouse). Data for two control clones (black squares) and the two AM-overexpressing clones (black triangles) were grouped together for the analysis. *P < 0.05, **P < 0.01, ***P < 0.001. **(D)** *Osteoclast formation in vitro* was unchanged by AM or 20–amino acid proadrenomedullin peptide (PAMP). D3 indicates the positive control, 1,25-dihydroxyvitamin D_3_. TRAP+, Tartrate-resistant acid phosphatase–positive. MNC, Multinucleated cells.

There are no reports that AM directly stimulates osteoclasts. We tested if AM or PAMP affected osteoclastogenesis in mouse bone marrow/osteoblast co-cultures. AM or PAMP peptide (10^−7^ M) did not increase TRAP-positive, osteoclast-like multinucleated cells compared to the untreated control (Figure 4A), whereas the positive control, 100 nM 1,25-dihydroxyvitamin D_3_, significantly increased TRAP-positive multinucleated cells. These results have been confirmed by others [19]. AM could increase early osteoblasts during the development of bone lesions [17],[18] because cells in the osteoblastic lineage control osteoclasts, which are responsible for increased bone destruction. This regulation of osteolysis is via osteoblastic expression of RANKL, the central regulator of osteoclast formation, survival and activity [40].

### Adrenomedullin overexpression increased MDA-MB-231 growth in mammary fat pads

AM overexpression caused larger MFP tumors compared to the GFP control clones (462 mm^3^ vs. 160 mm^3^ at day 21; *P* < 0.001) (Figure 4B). GFP control clones produced tumor volumes similar to each other, and AM-overexpressing clones also produced tumor volumes similar to each other (*P* = not significant). Martínez *et al*. also found that AM overexpression increased subcutaneous tumor growth of T47D breast cancer cells [37], although overexpression had the opposite effect in a prostate cancer model [41].

AM is proangiogenic, and many angiogenic factors, such as vascular endothelial growth factor and connective tissue growth factor, stimulate breast cancer bone metastases [20]. We compared the number of vessels in vascularized regions of MFP tumors using immunohistochemical staining for the endothelial cell marker CD31 (Figure 4C). The numbers of CD31-positive vessels were similar in the AM-overexpressing MFP tumors compared to controls (*P* = not significant) (Figure 4D). This was confirmed by staining for a second endothelial marker, von Willebrand factor (data not shown). MDA-MB-231 cells cause aggressive tumors in xenograft models and already express high levels of angiogenic factors; thus, increasing the expression of AM may have no additional effect on neoangiogenesis induced by this cell line.

Our data suggest that increased growth of AM-overexpressing MDA-MB-231 tumors is not due only to direct effects of AM on bone cells. AM is one of three vasoactive factors that increase the growth of breast cancer bone metastases. The vasorelaxant parathyroid hormone-related protein, PTHrP, increases osteolytic bone metastases, whereas the vasoconstrictor endothelin 1 (ET-1) stimulates osteoblastic metastases in breast cancer models [25],[27]. Vasoactive factors can alter tumor perfusion, and vasodilatation by AM increases tumor perfusion [42]. In the cases of PTHrP and ET-1, blockade of these factors decreases bone metastases without effects on MFP tumors [25],[27], indicating that factor actions on osteoblasts outweigh opposing effects on tumor perfusion. A major pathway by which AM may increase breast cancer growth in both MFP and bone may be via increased perfusion and oxygenation of the tumor. Future experiments are required to test this mechanism, but, at this point, the mechanisms by which AM overexpression increase growth in the MFP are unclear.

AM plays an important role in the growth and metastasis of many tumor types, where its transcription is increased by the hypoxia-response pathway, and it also stimulates bone cells. Our results establish that AM can accelerate bone metastasis in a standard breast cancer model. PTHrP expression by MDA-BM-231 cells dramatically increases osteolytic bone lesions [43] through actions on osteoblasts [44], cells which are stimulated by AM. The literature suggests that AM often exerts a general tumor-stimulatory effect. Our data show that it can increase bone metastases as well. AM is thus a potential drug target because it can stimulate cancer growth by autocrine effects on tumor plus paracrine effects on the microenvironment—a dual action that has been reported in pancreatic cancer [45].

### Small-molecule antagonists block adrenomedullin effects on bone cells

**Figure 5:**
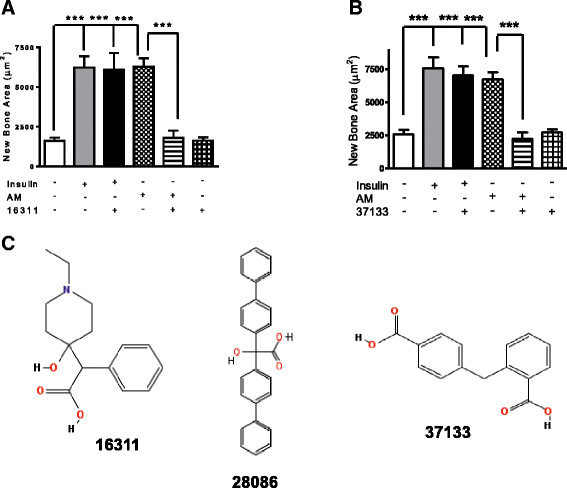
**Small-molecule antagonists block adrenomedullin-induced bone formation.** *Two small molecules blocked adrenomedullin (AM)-induced bone formation.* Neonatal mouse calvarial bones were cultured in triplicate for 7 days and treated with 10 nM AM ± 1 μM inhibitor NSC 16311 **(A)** or NSC 37133 **(B)**. Sections were assessed for new bone formation, expressed as area in histological sections, indicated on the vertical axis, by quantitative histomorphometry. ***P < 0.001. An example of the assay is provided in Additional file 1: Figure S1. **(C)** *Chemical structures* of the NSC compounds are shown [47].

AM inhibition could be a useful treatment for both primary tumors and bone metastases. Peptide antagonists [12],[14] successfully decrease primary tumor growth in mice, as do neutralizing antibodies [11],[13]. Such inhibitors could be used to reduce both primary tumor growth and osteolysis in breast cancer patients. Greater selectivity of inhibition may be obtained with small-molecule drugs that bind the circulating AM peptide and lock it into antagonist conformations [24],[46], which then suppress receptor-mediated responses. We tested three of the antagonists from an NCI library of compounds [47], studied by Martínez *et al*., for effects on bone in *ex vivo* calvarial organ cultures (Figure 5). The general methods of this assay are detailed elsewhere [30], and the response of neonatal bone to AM is shown in Additional file 1: Figure S1. The two effective ones, 16311 and 37133 (Figure 5C), are both simple aromatic carboxylic acids whose negative charge in solution may make them membrane-impermeant. The former has a dissociation constant for binding to AM of 8 nM without altered ligand–receptor association [24]. A third aromatic carboxylic acid, 28086 (Figure 5C), was ineffective in the assay (data not shown). We also tested 16311 and 37133 for effects on growth of primary osteoblasts isolated from neonatal mouse calvariae over a 48-hour period by MTT assay. There was no growth inhibition at concentrations from 10^−8^ to 10^−3^ M (Additional file 1: Figure S2). Their ability to block the bone-selective actions of AM and lack of cellular toxicity lends itself to further development of these small molecules for animal testing against breast cancer xenografts.

### Adrenomedullin antagonist 16311 decreases osteolytic factors TRAP and RANKL and growth of MDA-MB-231 cells in bone

The AM antagonist compounds [24] are untested against tumor growth *in vivo* because their pharmacokinetics and pharmacology for absorption, distribution, metabolism and excretion are unknown. We therefore studied one of them in a new *ex vivo* model of tumor–bone interaction [48] in which tumor cells are co-cultured for 1 week with neonatal calvarial bones. We have modified this procedure to resemble more closely the standard procedure for testing the effects of isolated factors on bone [30], using 5,000 MDA-MB-231 cells instead of 1 million. Representative images of the assay are provided in Additional file 1: Figure S3. The breast cancer cells were engineered stably to express a secreted *Cypridina* luciferase for noninvasive monitoring of tumor burden by assaying luciferase activity in the medium [32]. At the end of the incubation period, RNA was isolated from the co-cultures and assayed by species-specific real-time PCR [36] for changes in (human) tumor and (mouse) bone gene expression, an approach which yields more useful quantitative data than histology (Additional file 1: Figure S3).

**Figure 6:**
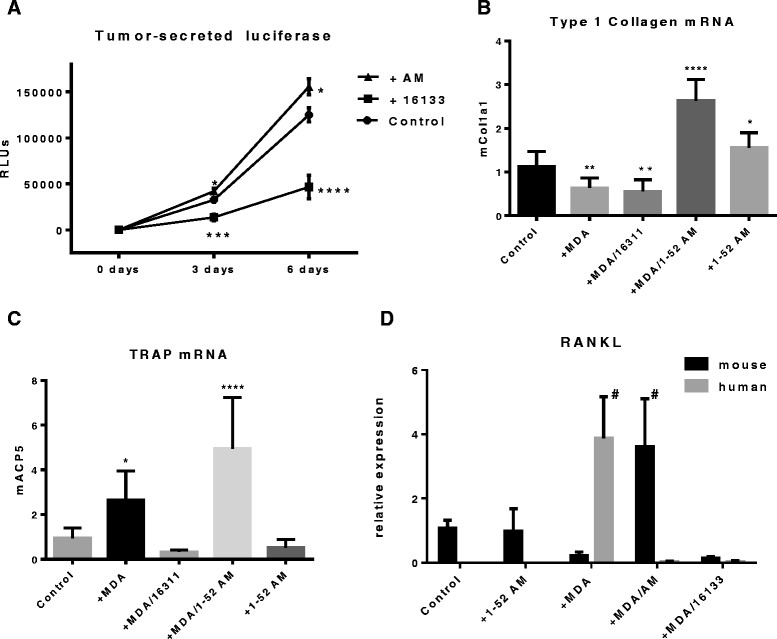
**Effects of adrenomedullin antagonist on MDA-MB-231 cells in bone.** All indicated *P*-values are versus control. **(A)** *Growth*. Addition of 10 nM adrenomedullin (AM) peptide (black triangles) increased tumor growth as measured by secreted luciferase marker in conditioned media. Significant vs control (*P < 0.05) at 3 and 6 days. Addition of 1 μM AM antagonist 16311 (black squares) dramatically decreased tumor growth vs control after 3 days (**P < 0.001) and 6 days (***P < 0.0001). RLU, Relative light unit. **(B)** *Osteoblast activity*. Bone biosynthetic collagen marker, type I collagen (mCol1A1). Exogenous AM increased mouse collagen mRNA in the presence and absence of tumor, whereas tumor alone (with or without 16311 antagonist) modestly suppressed this marker of osteoblast function. **(C)** *Osteoclast activity*. The mouse bone marker tartrate-resistant acid phosphatase (TRAP), encoded by mACP5, was increased by tumor cells, further increased by tumor plus exogenous AM and blocked by the 16311 AM antagonist. AM alone had no effect on TRAP mRNA. **(D)** Receptor activator of nuclear factor κB ligand (*RANKL)*. Samples were analyzed for both mouse and human RANKL expression because both tumor cells and bone cells can express this factor, which regulates osteoclast formation, survival and activity. Control is bone without tumor or treatment, where the expression of mouse RANKL = 1. Levels of human RANKL (gray bars) are adjusted to the equivalent threshold cycle values so that the absolute amounts of mouse and human RANKL are expressed on the same scale. In **(D)** only, # indicates significance relative to each of the other relevant columns within the mouse or human groups (^#^*P* < 0.0001) by one-way analysis of variance. No other pairwise comparisons were significantly different, and mouse and human datasets were not compared.

When MDA-MB-231 breast cancer cells were added to calvarial cultures, there was a substantial increase in tumor burden over the course of 6 days, as indicated by the secreted luciferase marker. Tumor activity was modestly but significantly further increased by supplementation with 10 nM AM peptide. In contrast, the addition of the 16311 antagonist to tumor/bone co-cultures dramatically decreased tumor burden in the absence of added AM (Figure 6A). Antagonist alone had no apparent effect on bone (Figure 5A). The species-specific PCR analysis of these cultures confirmed that AM (in the presence or absence of tumor) increased the marker of osteoblast activity, type 1 collagen, consistent with the stimulation of bone formation seen with AM [16] (Additional file 1: Figure S1). MDA-MB-231 cells alone (with or without the 16311 antagonist) caused modest suppression of collagen mRNA (Figure 6B). To interrogate the mechanism underlying the tumor–bone interactions altered by AM, we looked at markers of osteoclast activity and osteoclast regulation because increased osteolytic bone destruction is a hallmark of breast cancer bone metastases and a central contributor to the vicious cycle [20]. Osteoclast formation is controlled by RANKL, which regulates osteoclast formation, survival and activity. Its action is opposed by a soluble binding protein, osteoprotegerin (Opg). Both proteins are made by cells of the osteoblastic lineage. Active osteoclasts destroy bone through the controlled secretion of acid and enzymes, in particular TRAP and cathepsin K. We found that adding MDA-MB-231 cells to bone increased TRAP expression. AM alone had no effect, whereas the addition of AM to cultures containing breast cancer cells further increased bone expression of TRAP mRNA. Induction of TRAP by breast cancer cells was completely blocked by the addition of 1 μM 16311 AM antagonist (Figure 6C). The *P*-values indicated in Figure 6C are all relative to control (bone without tumor). In addition, when columns 2 and 4 were compared (bone with tumor with or without exogenous AM) by one-way ANOVA, the addition of 1 nM AM significantly augmented TRAP mRNA (*P* < 0.01). Responses similar to those seen with TRAP were also observed for cathepsin K (Additional file 1: Figure S4A). We had previously observed a dramatic increase in expression of RANKL mRNA by MDA-MB-231 cells grown in bone, and such expression has been reported by others [49]. Therefore, we assayed for tumor and bone expression of RANKL, both of which are readily distinguished by PCR with species-specific primers. Figure 6D shows that AM alone does not alter RANKL expression, consistent with its lack of effect on osteoclasts. MDA-MB-231 cells alone suppress mouse RANKL mRNA, whereas the presence of bone induces tumor expression of human RANKL. Addition of 10 nM AM to the system reverses the expression pattern, so that mouse bone RANKL is abundant and human tumor RANKL is low. Both responses in RANKL were statistically significant. The addition of 16311 antagonist blocked expression of RANKL from both sources. The RANKL-neutralizing protein Opg was very low in all groups (Additional file 1: Figure S4B). An early discovery of a vicious cycle in breast cancer bone metastases was with the osteolytic factor PTHrP, which is made by MDA-MB-231 cells and acts indirectly by increasing stromal RANKL expression [44]. We found that tumor expression of PTHrP in the presence of bone was unchanged by treatment with AM or the 16311 antagonist (Additional file 1: Figure S4C). Thus, the changes in RANKL and in osteoclast markers that we observed were not responses to changes in PTHrP. Overall, the results show that AM increases tumor-induced osteolysis in bone by altering local production of RANKL. This change, as well as tumor growth, could be blocked with the 16311 antagonist.

Our data (Figure 4D), as well as results published elsewhere by others [19], show that simple addition of AM peptide to bone cultures does not increase osteoclasts. How, then, does AM in the tumor microenvironment accelerate osteolytic bone metastases? AM stimulates osteoblasts and bone formation, and cells in the osteoblast lineage regulate osteoclasts via the RANKL pathway. RANKL is expressed by bone stromal cells and by osteocytes, cells early and late in the osteoblast lineage, where a variety of tumor-secreted products increase RANKL expression [50]. AM could increase the number of RANKL-expressing cells or potentiate their production of RANKL in response to osteolytic factors such as IL-6 or PTHrP. The data in Figure 6D show that the addition of tumor cells resulted in a fourfold increase in bone RANKL mRNA, a response that is abrogated by adding AM antagonist. These results are consistent with the changes seen in the osteoclast marker TRAP mRNA in Figure 6C. AM retained its ability in these co-cultures to stimulate the osteoblast marker type I collagen (Figure 6B), regardless of the presence of tumor cells. The dramatic switch in the source of RANKL expression (from tumor to bone) upon addition of a high dose of AM peptide, shown in Figure 6D, was unexpected and remains without a compelling explanation. Osteocytes are a major source of RANKL [40], but the effects of AM on these cells are not known. Tumor cells can also be a source of RANKL in osteolytic lesions [51]. RANKL decreases AM receptor signaling via alterations in cAMP [19]. So, interactions between signaling pathways involving AM could contribute to the switch in cellular source of RANKL. In recent studies with the MDA-MB-231 model of bone metastases, researchers found that crosstalk between tumor and osteoblastic cells increased metastatic growth via a subsidiary vicious cycle in which IL-6 and RANKL increased one another in the metastatic microenvironment [52]. AM can increase IL-6 expression in mesenchymal cells, but this has not been shown with osteoblasts or cancer cells. The role of AM in regulation of RANKL expression in bone metastases is a subject of ongoing investigation.

## Conclusions

Tumor-expressed AM contributes to the growth of breast cancer cells as osteolytic metastases in bone. The general phenotype of the bone lesions was unaltered by increased AM, but the rate of their growth was increased and was accompanied by shorter survival. AM overexpression also increased growth of tumor in the MFP, but not *in vitro*, in the absence of increased angiogenesis, by unknown mechanisms. Our results with overexpression of the AM peptide in a standard animal model, combined with experiments in *ex vivo* co-cultures of cancer cells with bone, provide the first proof of principle that AM may be a useful target for the treatment of breast cancer bone metastasis. A small-molecule inhibitor blocked bone responses to AM *ex vivo* without general cellular toxicity and decreased osteoclast markers and tumor growth in bone. Our data support preclinical development of AM antagonists for *in vivo* testing and future treatment of osteolytic metastases due to breast cancer.

## Authors’ information

The research described was carried out as part of the dissertation project of VAS for the doctoral degree in biochemistry at the University of Virginia.

## Additional file

## Electronic supplementary material

Additional file 1: Supplemental material. (PDF 615 KB)

Below are the links to the authors’ original submitted files for images.

Authors’ original file for figure 1

Authors’ original file for figure 2

Authors’ original file for figure 3

Authors’ original file for figure 4

Authors’ original file for figure 5

Authors’ original file for figure 6

## Abbreviations

AM: Adrenomedullin (1–52) amide
ANOVA: Analysis of variance
CGRP: Calcitonin gene-related peptide
CMV: Cytomegalovirus
DMEM: Dulbecco’s modified Eagle’s medium
emGFP: Emerald green fluorescent protein
ET-1: Endothelin-1
FBS: Fetal bovine serum
IL: Interleukin
GFP: Green fluorescent protein
H&E: Hematoxylin and eosin
IRES: Internal ribosome entry site
MFP: Mammary fat pad
MTT: 3-(4,5-dimethylthiazol-2-yl)-2,5-diphenyltetrazolium bromide
NCBI: National Center for Biotechnology Information
Opg: Osteoprotegerin
PAMP: Proadrenomedullin peptide (20 amino acids)
PBS: Phosphate-buffered saline
PTHrP: Parathyroid hormone-related peptide
RAMP: Receptor activity–modifying protein
RANKL: Receptor activator of nuclear factor κB ligand
RFP: Red fluorescent protein
TRAP: Tartrate-resistant acid phosphatase

## References

[1] Eto T (2001). A review of the biological properties and clinical implications of adrenomedullin and proadrenomedullin N-terminal 20 peptide (PAMP), hypotensive and vasodilating peptides. Peptides.

[2] Garayoa M, Martínez A, Lee S, Pío R, An WG, Neckers L, Montuenga LM, Ryan H, Johnson R, Gassmann M, Cuttitta F (2000). Hypoxia-inducible factor-1 (HIF-1) up-regulates adrenomedullin expression in human tumor cell lines during oxygen deprivation: a possible promotion mechanism of carcinogenesis. Mol Endocrinol.

[3] Pio R, Martinez A, Unsworth EJ, Kowalak JA, Bengoechea JA, Zipfel PF, Elsasser TH, Cuttitta F (2001). Complement factor H is a serum-binding protein for adrenomedullin, and the resulting complex modulates the bioactivities of both partners. J Biol Chem.

[4] Kuwasako K, Kitamura K, Nagata S, Hikosaka T, Takei Y, Kato J (2011). Shared and separate functions of the RAMP-based adrenomedullin receptors. Peptides.

[5] Bunton DC, Petrie MC, Hillier C, Johnston F, McMurray JJ (2004). The clinical relevance of adrenomedullin: a promising profile?. Pharmacol Ther.

[6] Miller MJ, Martínez A, Unsworth EJ, Thiele CJ, Moody TW, Elsasser T, Cuttitta F (1996). Adrenomedullin expression in human tumor cell lines: its potential role as an autocrine growth factor. J Biol Chem.

[7] Zudaire E, Martínez A, Cuttitta F (2003). Adrenomedullin and cancer. Regul Pept.

[8] Hay DL, Walker CS, Poyner DR (2011). Adrenomedullin and calcitonin gene-related peptide receptors in endocrine-related cancers: opportunities and challenges. Endocr Relat Cancer.

[9] Oehler MK, Fischer DC, Orlowska-Volk M, Herrle F, Kieback DG, Rees MC, Bicknell R (2003). Tissue and plasma expression of the angiogenic peptide adrenomedullin in breast cancer. Br J Cancer.

[10] Ribatti D, Nico B, Spinazzi R, Vacca A, Nussdorfer GG (2005). The role of adrenomedullin in angiogenesis. Peptides.

[11] Ouafik L, Sauze S, Boudouresque F, Chinot O, Delfino C, Fina F, Vuaroqueaux V, Dussert C, Palmari J, Dufour H, Grisoli F, Casellas P, Brünner N, Martin PM (2002). Neutralization of adrenomedullin inhibits the growth of human glioblastoma cell lines *in vitro* and suppresses tumor xenograft growth *in vivo*. Am J Pathol.

[12] Miseki T, Kawakami H, Natsuizaka M, Darmanin S, Cui HY, Chen J, Fu Q, Okada F, Shindo M, Higashino F, Asaka M, Hamuro J, Kobayashi M (2007). Suppression of tumor growth by intra-muscular transfer of naked DNA encoding adrenomedullin antagonist. Cancer Gene Ther.

[13] Kaafarani I, Fernandez-Sauze S, Berenguer C, Chinot O, Delfino C, Dussert C, Metellus P, Boudouresque F, Mabrouk K, Grisoli F, Figarella-Branger D, Martin PM, Ouafik L (2009). Targeting adrenomedullin receptors with systemic delivery of neutralizing antibodies inhibits tumor angiogenesis and suppresses growth of human tumor xenografts in mice. FASEB J.

[14] Ishikawa T, Chen J, Wang J, Okada F, Sugiyama T, Kobayashi T, Shindo M, Higashino F, Katoh H, Asaka M, Kondo T, Hosokawa M, Kobayashi M (2003). Adrenomedullin antagonist suppresses *in vivo*growth of human pancreatic cancer cells in SCID mice by suppressing angiogenesis. Oncogene.

[15] Naot D, Cornish J (2008). The role of peptides and receptors of the calcitonin family in the regulation of bone metabolism. Bone.

[16] Cornish J, Naot D, Reid IR (2003). Adrenomedullin—a regulator of bone formation. Regul Pept.

[17] Cornish J, Callon KE, Coy DH, Jiang NY, Xiao L, Cooper GJ, Reid IR (1997). Adrenomedullin is a potent stimulator of osteoblastic activity in vitro and *in vivo*. Am J Physiol.

[18] Cornish J, Callon KE, Bava U, Coy DH, Mulvey TB, Murray MA, Cooper GJ, Cooper GJ, Reid IR (2001). Systemic administration of adrenomedullin(27–52) increases bone volume and strength in male mice. J Endocrinol.

[19] Granholm S, Henning P, Lerner UH (2011). Comparisons between the effects of calcitonin receptor-stimulating peptide and intermedin and other peptides in the calcitonin family on bone resorption and osteoclastogenesis. J Cell Biochem.

[20] Siclari VA, Guise TA, Chirgwin JM (2006). Molecular interactions between breast cancer cells and the bone microenvironment drive skeletal metastases. Cancer Metastasis Rev.

[21] Dai X, Ma W, Jha RK, He X (2010). Adrenomedullin and its expression in cancers and bone: a literature review. Front Biosci (Elite Ed).

[22] Tubiana-Hulin M (1991). Incidence, prevalence and distribution of bone metastases. Bone.

[23] Coleman RE (2001). Metastatic bone disease: clinical features, pathophysiology and treatment strategies. Cancer Treat Rev.

[24] Martínez A, Julián M, Bregonzio C, Notari L, Moody TW, Cuttitta F (2004). Identification of vasoactive nonpeptidic positive and negative modulators of adrenomedullin using a neutralizing antibody-based screening strategy. Endocrinology.

[25] Yin JJ, Selander K, Chirgwin JM, Dallas M, Grubbs BG, Wieser R, Massagué J, Mundy GR, Guise TA (1999). TGF-β signaling blockade inhibits PTHrP secretion by breast cancer cells and bone metastases development. J Clin Invest.

[26] Lu X, Kang Y (2009). Efficient acquisition of dual metastasis organotropism to bone and lung through stable spontaneous fusion between MDA-MB-231 variants. Proc Natl Acad Sci U S A.

[27] Yin JJ, Mohammad KS, Kakonen SM, Harris S, Wu-Wong JR, Wessale JL, Padley RJ, Garrett IR, Chirgwin JM, Guise TA (2003). A causal role for endothelin-1 in the pathogenesis of osteoblastic bone metastases. Proc Natl Acad Sci U S A.

[28] Choi WW, Lewis MM, Lawson D, Yin-Goen Q, Birdsong GG, Cotsonis GA, Cohen C, Young AN (2005). Angiogenic and lymphangiogenic microvessel density in breast carcinoma: correlation with clinicopathologic parameters and VEGF-family gene expression. Mod Pathol.

[29] Takahashi N, Akatsu T, Udagawa N, Sasaki T, Yamaguchi A, Moseley JM, Martin TJ, Suda T (1988). Osteoblastic cells are involved in osteoclast formation. Endocrinology.

[30] Mohammad KS, Chirgwin JM, Guise TA (2008). Assessing new bone formation in neonatal calvarial organ cultures. Methods Mol Biol.

[31] Bakker A, Klein-Nulend J (2003). Osteoblast isolation from murine calvariae and long bones. Methods Mol Med.

[32] Tannous BA, Teng (2011). Secreted blood reporters: insights and applications. Biotechnol Adv.

[33] Yuan JS, Wang D, Stewart CN (2008). Statistical methods for efficiency adjusted real-time PCR quantification. Biotechnol J.

[34] Untergasser A, Cutcutache I, Koressaar T, Ye J, Faircloth BC, Remm M, Rozen SG (2012). Primer3—new capabilities and interfaces. Nucleic Acids Res.

[35] Ye J, Coulouris G, Zaretskaya I, Cutcutache I, Rozen S, Madden TL (2012). Primer-BLAST: a tool to design target-specific primers for polymerase chain reaction. BMC Bioinformatics.

[36] Drew AF, Blick TJ, Lafleur MA, Tim EL, Robbie MJ, Rice GE, Quinn MA, Thompson EW (2004). Correlation of tumor- and stromal-derived MT1-MMP expression with progression of human ovarian tumors in SCID mice. Gynecol Oncol.

[37] Martinez A, Vos M, Guedez L, Kaur G, Chen Z, Garayoa M, Pío R, Moody T, Stetler-Stevenson WG, Kleinman HK, Cuttitta F (2002). The effects of adrenomedullin overexpression in breast tumor cells. J Natl Cancer Inst.

[38] Mundy GR (2001). Preclinical models of bone metastases. Semin Oncol.

[39] Kang Y, Siegel PM, Shu W, Drobnjak M, Kakonen SM, Cordon-Cardo C, Guise TA, Massagué J (2003). A multigenic program mediating breast cancer metastasis to bone. Cancer Cell.

[40] O’Brien CA, Nakashima T, Takayanagi H (2013). Osteocyte control of osteoclastogenesis. Bone.

[41] Abasolo I, Wang Z, Montuenga LM, Calvo A (2004). Adrenomedullin inhibits prostate cancer cell proliferation through a cAMP-independent autocrine mechanism. Biochem Biophys Res Commun.

[42] Iimuro S, Shindo T, Moriyama N, Amaki T, Niu P, Takeda N, Iwata H, Zhang Y, Ebihara A, Nagai R (2004). Angiogenic effects of adrenomedullin in ischemia and tumor growth. Circ Res.

[43] Kakonen SM, Selander KS, Chirgwin JM, Yin JJ, Burns S, Rankin WA, Grubbs BG, Dallas M, Cui Y, Guise TA (2002). Transforming growth factor-β stimulates parathyroid hormone-related protein and osteolytic metastases via Smad and mitogen-activated protein kinase signaling pathways. J Biol Chem.

[44] Thomas RJ, Guise TA, Yin JJ, Elliott J, Horwood NJ, Martin TJ, Gillespie MT (1999). Breast cancer cells interact with osteoblasts to support osteoclast formation. Endocrinology.

[45] Ramachandran V, Arumugam T, Langley R, Hwang RF, Vivas-Mejia P, Sood AK, Lopez-Berestein G, Logsdon CD (2009). The ADMR receptor mediates the effects of adrenomedullin on pancreatic cancer cells and on cells of the tumor microenvironment. PLoS One.

[46] Julián M, Cacho M, García MA, Martín-Santamaría S, de Pascual-Teresa B, Ramos A, Martínez A, Cuttitta F (2005). Adrenomedullin: a new target for the design of small molecule modulators with promising pharmacological activities. Eur J Med Chem.

[47] CADD Group Chemoinformatics Tools and User Services [: Enhanced NCI Database Browser Release 2.2 [http://cactus.nci.nih.gov/ncidb2.2/], [http://cactus.nci.nih.gov/index.html]

[48] Curtin P, Youm H, Salih E (2012). Three-dimensional cancer-bone metastasis model using ex-vivo co-cultures of live calvarial bones and cancer cells. Biomaterials.

[49] Tang ZN, Zhang F, Tang P, Qi XW, Jiang J (2011). Hypoxia induces RANK and RANKL expression by activating HIF-1α in breast cancer cells. Biochem Biophys Res Commun.

[50] Roodman GD, Dougall WC (2008). RANK ligand as a therapeutic target for bone metastases and multiple myeloma. Cancer Treat Rev.

[51] Lai FP, Cole-Sinclair M, Cheng WJ, Quinn JM, Gillespie MT, Sentry JW, Schneider HG (2004). Myeloma cells can directly contribute to the pool of RANKL in bone bypassing the classic stromal and osteoblast pathway of osteoclast stimulation. Br J Haematol.

[52] Zheng Y, Chow SO, Boernert K, Basel D, Mikuscheva A, Kim S, Fong-Yee C, Trivedi T, Buttgereit F, Sutherland RL, Dunstan CR, Zhou H, Seibel MJ (2014). Direct crosstalk between cancer and osteoblast lineage cells fuels metastatic growth in bone via auto-amplification of IL-6 and RANKL signaling pathways. J Bone Miner Res.

