# Supplementary material for: Tumor-expressed adrenomedullin accelerates breast cancer bone metastasis
Source: Breast Cancer Res. 2014 Dec 2;16:458. doi: 10.1186/s13058-014-0458-y (PMC4303191; doi:10.1186/s13058-014-0458-y)
Supplement: Supplementary file 1 — Additional file 1: Supplemental material. (PDF 615 KB) [file 13058_2014_458_MOESM1_ESM.pdf]

Supplemental Material

Specific PCR primers were designed as detailed in the main text:

Mouse TRAP: mAcp5 (forward): TTCAGGACGAGAACGGTGTG; (reverse): CTCTCGTGGTGTTCAGGGTC

Mouse type 1 collagen: mCol1A1 (forward): GTGTGCGATGACGTGCAATG; (reverse): TTGGGTCCCTCGACTCCTAC

Mouse cathepsin K: mCtsK (forward): CTTCCAATACGTGCAGCAGA; (reverse): TTGCATCGATGGACACAGAG

Human PTHrP: hPTHLH (forward): AACACAAAGAACCACCCCGT; (reverse): GTGGAGGTGTCAGACAGGTG

Mouse RPL32: mRPL32 (forward): GCTGCCATCTGTTTTACGGC; (reverse); CGTTGGGATTGGTGACTCTGA

Human RANKL: hTNFSF1 (forward): GGTTGGGCCAAGATCTCCAA; (reverse): TCCGGATCCAGTAAGGAGGG

Mouse RANKL: mTNFSF11(forward): AAAACGCAGGTTTGCAGGAC; (reverse): GTGAGGTGTGCAAATGGCTG

Mouse Opg: mTNFRSF11b (forward): TGAAAGCGTTAACCCTGGAGC; (reverse): GAAACCTCAGGGGCTTGGAG

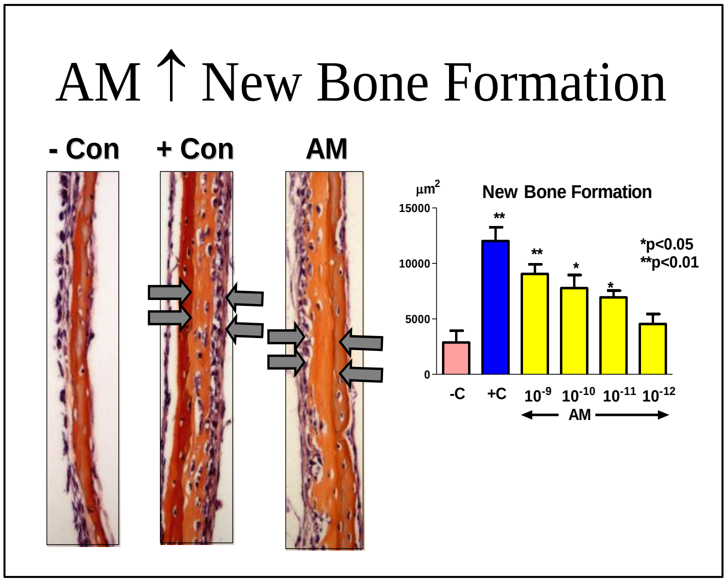

Figure S1: <u>Stimulation of new bone formation by adrenomedullin</u>. AM1-52amide peptide was added to the neonatal calvarial assay for 7d as described by Mohammad et al [31] with n=3. Bones were fixed, decalcified and stained with H&E. New bone area (left) was scored as orange area (between gray arrows). Older bone stains darker red. Positive control is 100nM insulin. New bone area was quantified by computerized histo-morphometry and results shown on right.

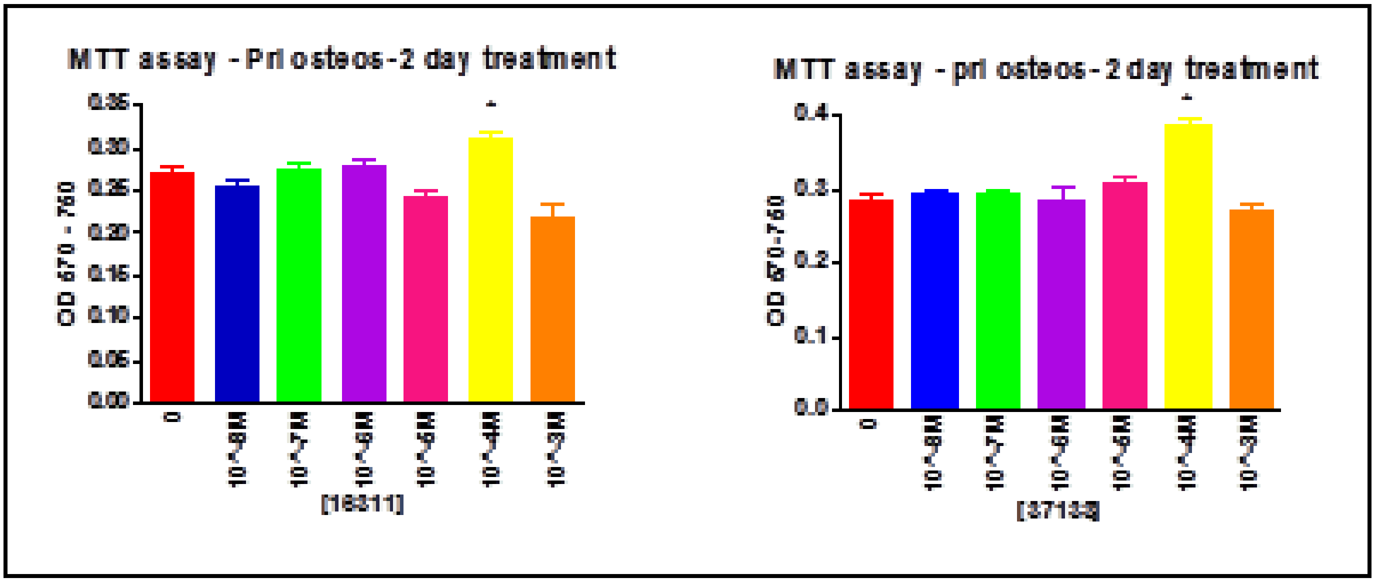

Figure S2: <u>Cellular toxicity of 16311 & 37133</u>. Primary osteoblasts (Pri osteos) treated with 16311 or 37133 for 2d and cells counted by MTT assay. OD measured at 570nm & 750nm for normalization. No toxicity seen with 2d of treatment at up to 1mM. There was slightly increased proliferation at 10^{-4}M 16311 or 37133 ($p<0.05$ by one-way ANOVA).

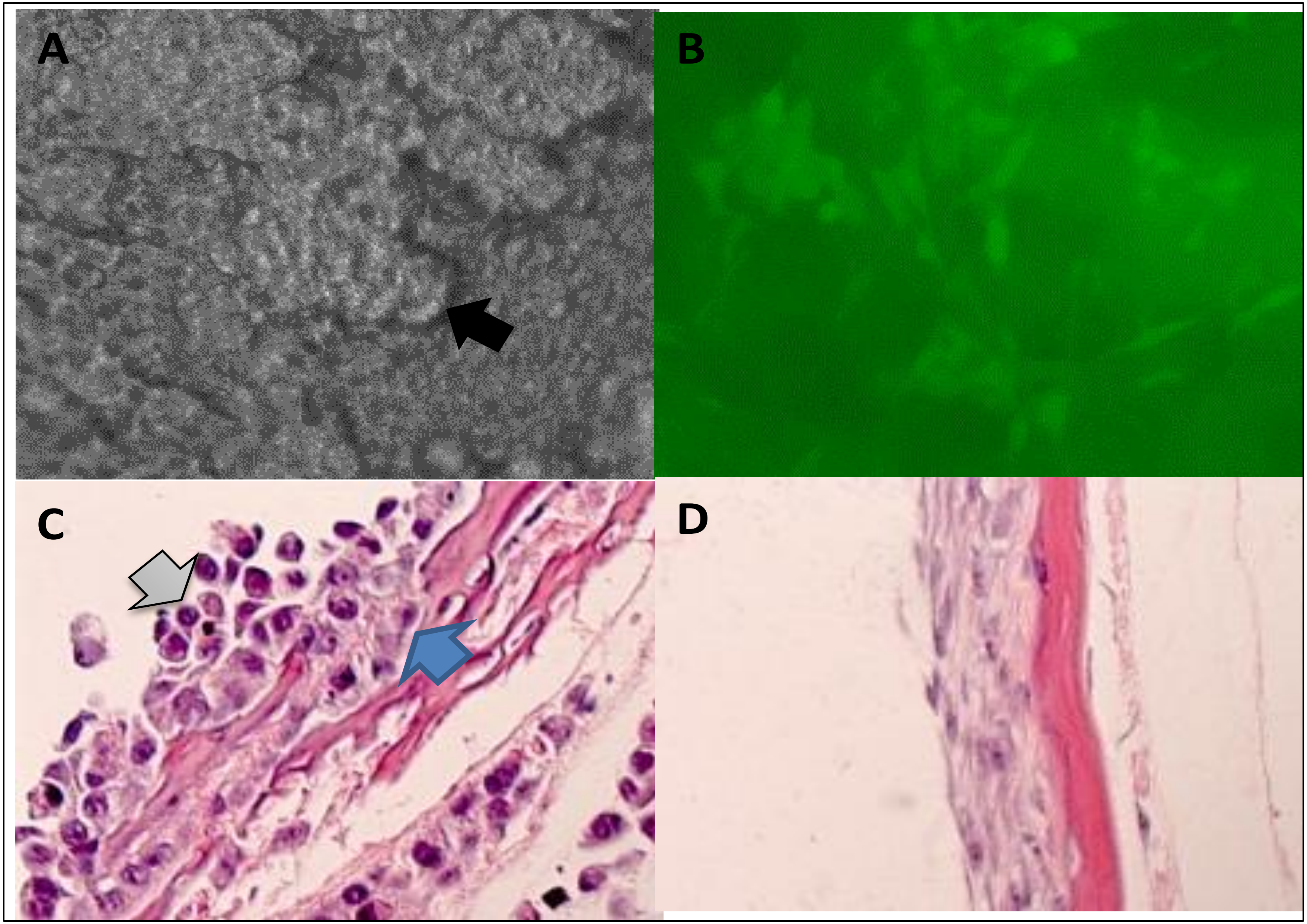

Figure S3: MDA-MB-231 cells grown 7d with mouse calvariae. (**A**) Phase contrast: lucent lesions with scalloping edges (**arrow**). (**B**) Fluorescence of GFP cells in clusters. (**C**) H&E: tumor cells in bone (**gray arrow**) causing osteolysis (**blue arrow**), vs control bone (**D**).

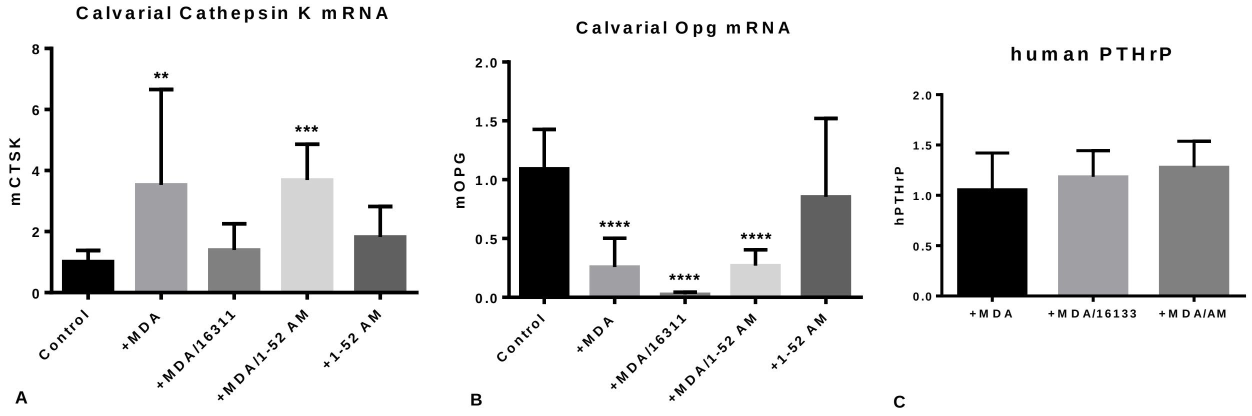

Figure S4: Changes in gene expression in tumor:bone co-cultures. Additional analyses of RNA samples from main Figure 6. (**A**) Osteoclast marker cathepsin K (mCTSK) gave results similar to TRAP in Figure 6C. (**B**) Negative regulator of osteoclast formation, osteoprotegerin (Opg = mTNFRSF11B), which binds and neutralizes RANKL. All groups expressed only low levels of Opg mRNA with C_t values >30. (**C**) Osteolytic factor PTHrP (hPTHLP) was unchanged in any of the groups. It is expressed by MDA-MB-231 breast cancer cells [25], is increased by TGFbeta in bone [41] and acts on osteoblasts to increase osteolysis by stimulating RANKL expression [42].
