# Supplementary figures and images for: Tumor-expressed adrenomedullin accelerates breast cancer bone metastasis
Source: Breast Cancer Res. 2014 Dec 2;16:458. doi: 10.1186/s13058-014-0458-y (PMC4303191; doi:10.1186/s13058-014-0458-y)

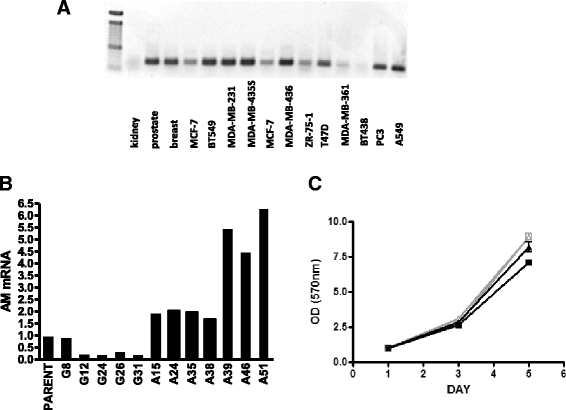

Supplement: Supplementary file 2 — Authors’ original file for figure 1 [file 13058_2014_458_MOESM2_ESM.gif]

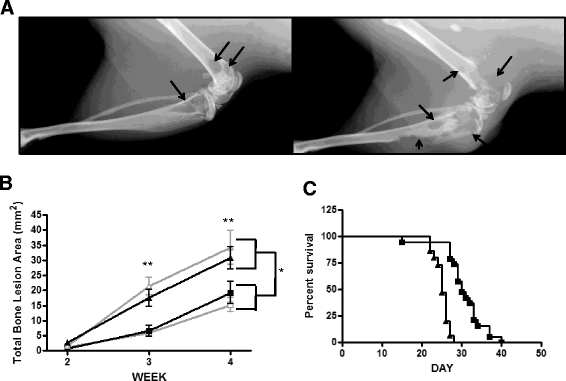

Supplement: Supplementary file 3 — Authors’ original file for figure 2 [file 13058_2014_458_MOESM3_ESM.gif]

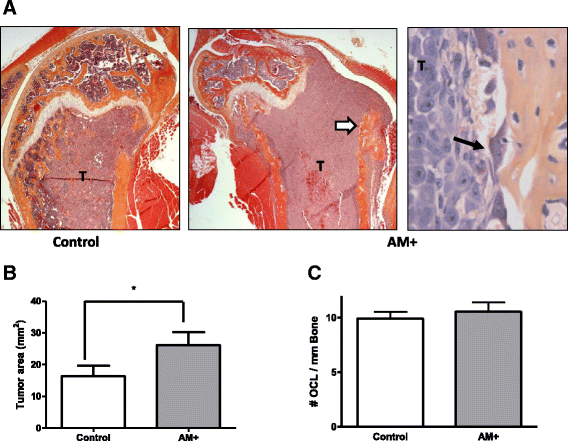

Supplement: Supplementary file 4 — Authors’ original file for figure 3 [file 13058_2014_458_MOESM4_ESM.gif]

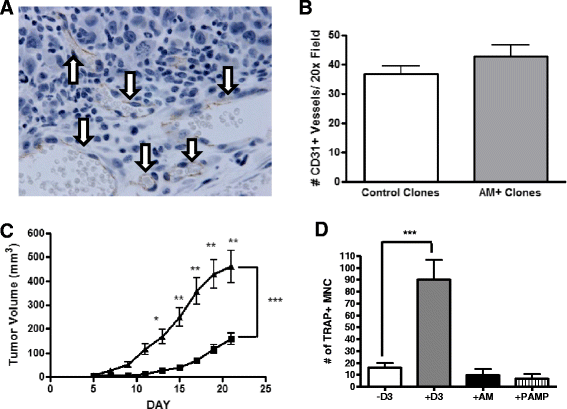

Supplement: Supplementary file 5 — Authors’ original file for figure 4 [file 13058_2014_458_MOESM5_ESM.gif]

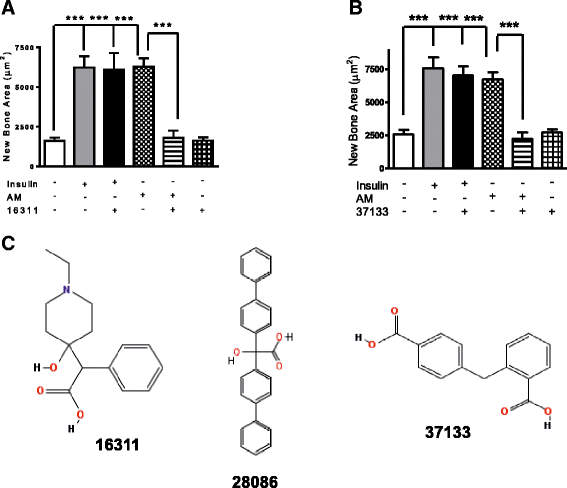

Supplement: Supplementary file 6 — Authors’ original file for figure 5 [file 13058_2014_458_MOESM6_ESM.gif]

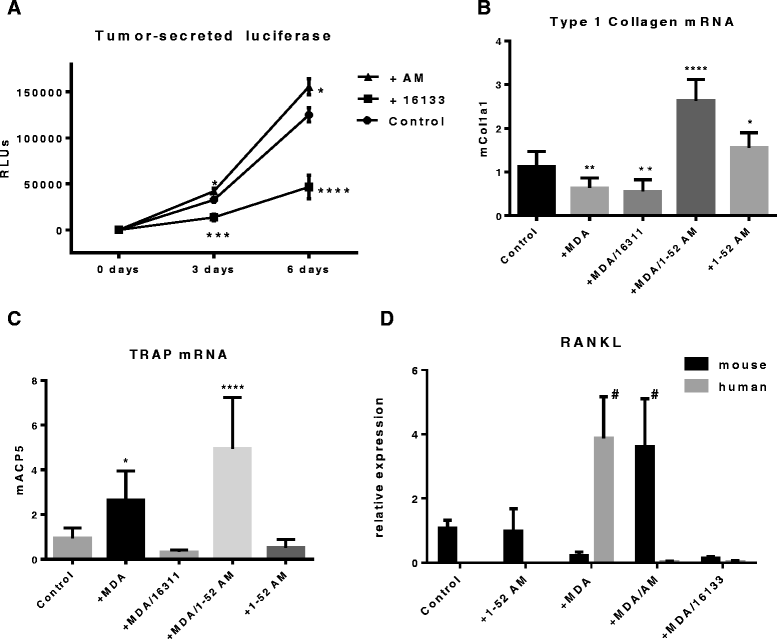

Supplement: Supplementary file 7 — Authors’ original file for figure 6 [file 13058_2014_458_MOESM7_ESM.gif]
